# Supplementary material for: Structural insights into the assembly of gp130 family cytokine signaling complexes
Source: Sci Adv. 2023 Mar 17;9(11):eade4395. doi: 10.1126/sciadv.ade4395 (PMC10022904; doi:10.1126/sciadv.ade4395)
Supplement: Supplementary file 1 — Figs. S1 to S10 Tables S1 and S2 [file sciadv.ade4395_sm.pdf]

Science Advances
AAAS

Supplementary Materials for

Structural insights into the assembly of gp130 family cytokine signaling complexes

Yi Zhou *et al.*

Corresponding author: Yi Zhou, yi.zhou@regeneron.com; Matthew C. Franklin, matthew.franklin@regeneron.com

Sci. Adv. **9**, eade4395 (2023)
DOI: 10.1126/sciadv.ade4395

This PDF file includes:

Figs. S1 to S10
Tables S1 and S2

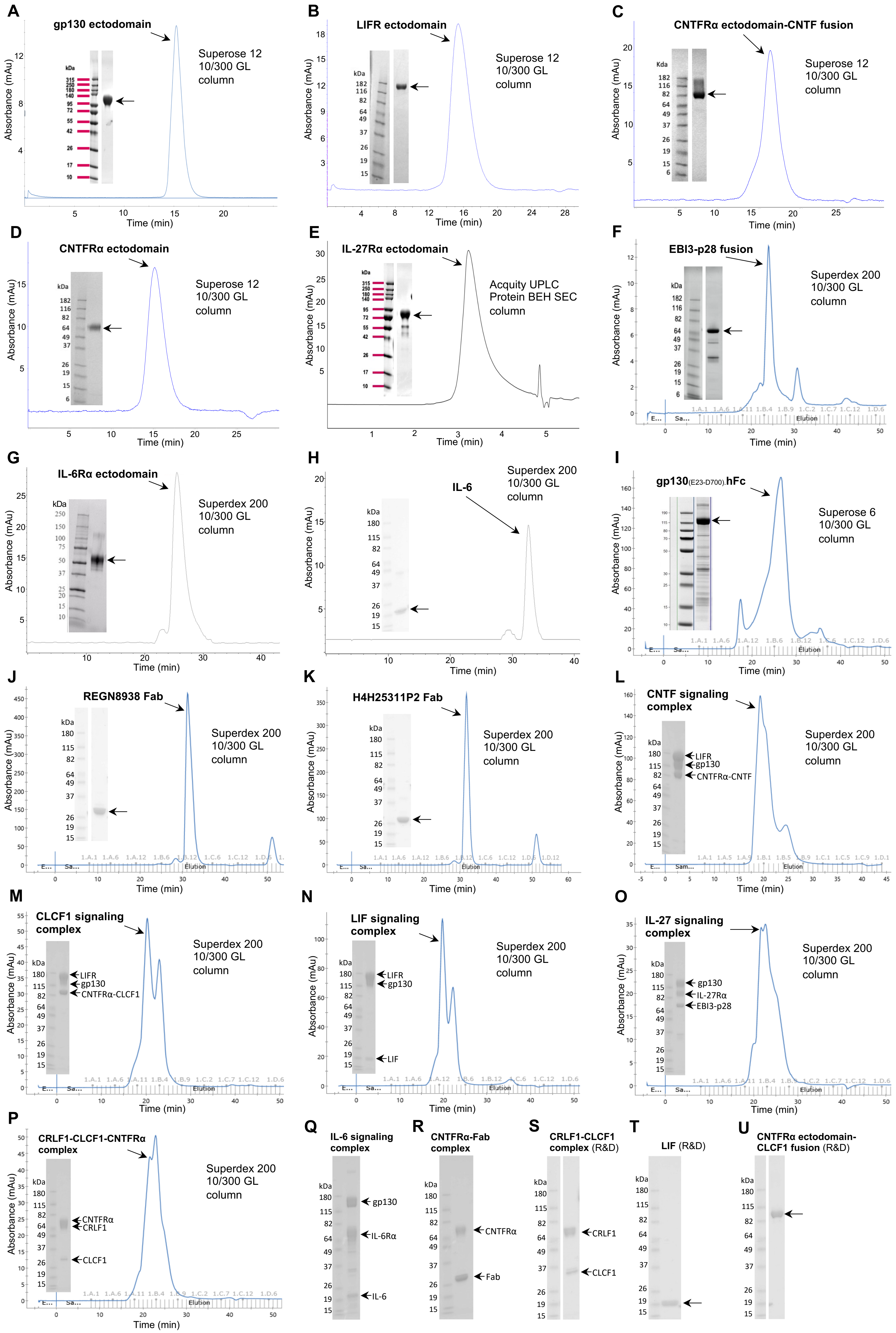

Fig. S1. Size-exclusion chromatography (SEC) and SDS-PAGE (reducing) analysis of proteins and proteins complexes used in this study

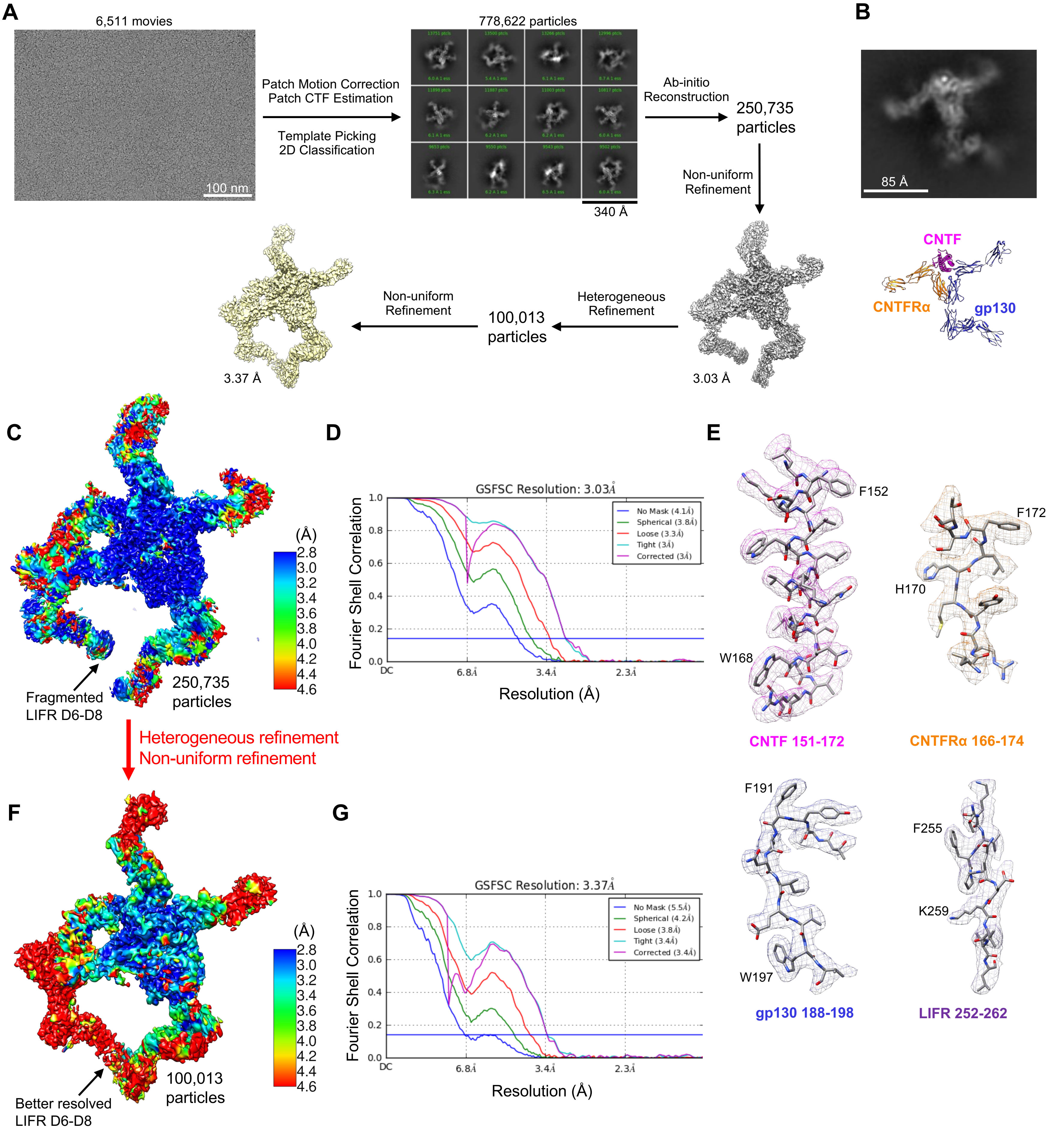

Fig. S2. Cryo-EM analysis of the CNTF signaling complex

(A) Workflow for cryo-EM data processing.
(B) CNTF complex intermediate (CNTF/CNTFRα/gp130 sub-complex) observed on the EM grid.
(C) Local resolution estimation of the final cryo-EM density map showing around 2.8 Å local resolution at the interaction core region. This map was used for building the atomic model of the interaction core region, including CNTF, CNTFRα D2D3, gp130 D2-D5, and LIFR D2-D5. The model was used to analyze the interaction details as shown in Figs. 2A-2D. However, the map has fragmented LIFR D6-D8 density due to flexibility-induced heterogeneity of this region.
(D) FSC curve of the CNTF complex reconstruction showing a global resolution of 3.03 Å with the 0.143 gold standard threshold.
(E) Representative cryo-EM density of the 3D reconstruction in (C).
(F) The 250,735 particles from (C) were further subjected to heterogeneous refinement, identifying 100,013 particles with more homogeneous LIFR D6-D8. Non-uniform refinement of this subset of particles yielded a map with better density at LIFR FNIII domains. The local resolution of the map was estimated, showing lower global resolution (3.37 Å). This map was used to generate a complete model of the CNTF complex with full ECDs by combining the structure of the interaction core region and models of the receptor distal domains derived from published structures (gp130 D1, PDB: 1P9M; gp130 D6, PDB: 3L5H; LIFR D1, PDB:3E0G), a structure obtained in this study (CNTFRα D1, fig. S3), and a model predicted by AlphaFold (LIFR D6-D8). The map and model were used to show the overall architecture of the complex as shown in Figs. 1A-1C.
(G) FSC curve of the CNTF complex reconstruction in (F) showing a global resolution of 3.37 Å with the 0.143 gold standard threshold.

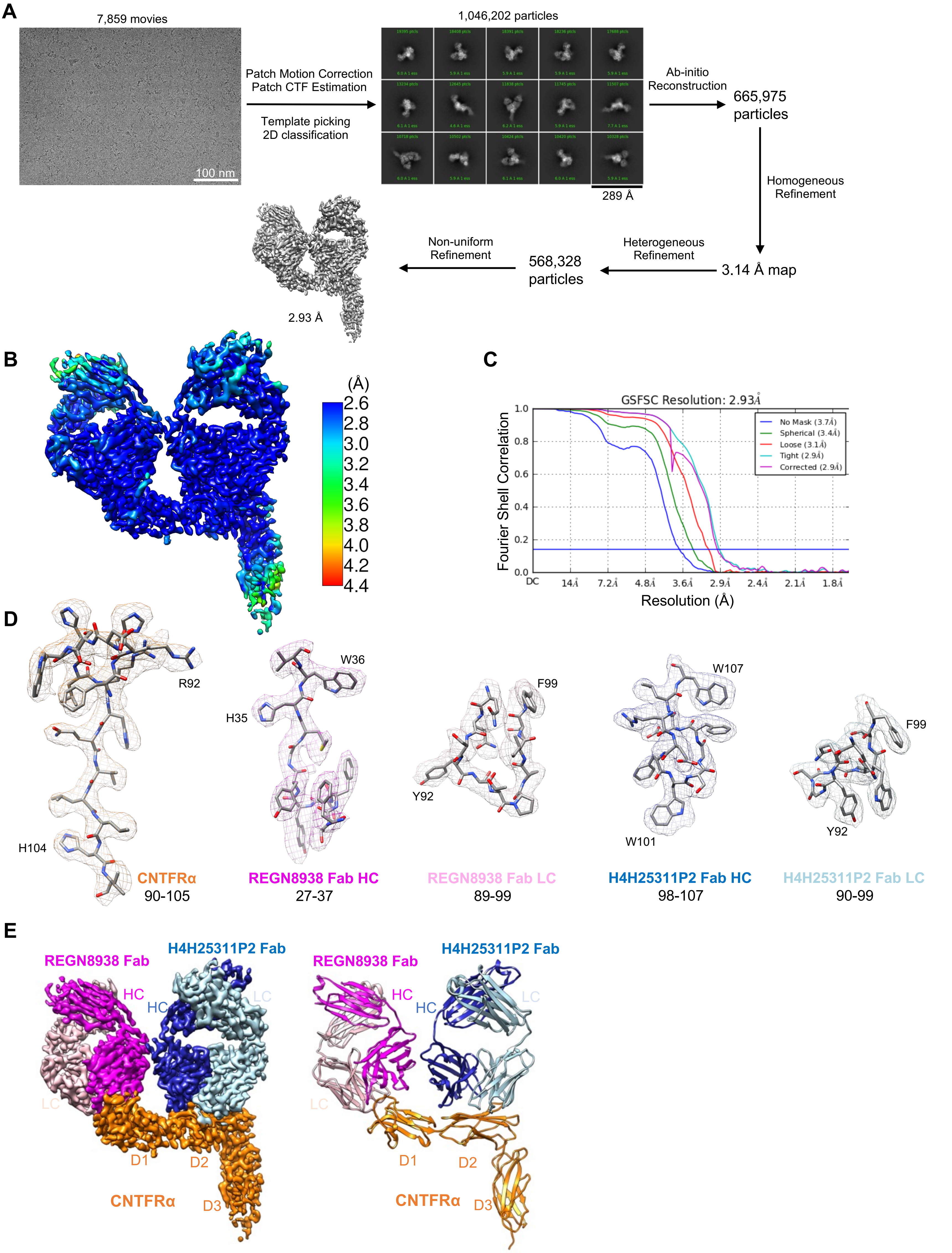

Fig. S3. Cryo-EM structure of CNTFRα in complex with REGN8938 Fab and H4H25322P2 Fab

(A) Workflow for cryo-EM data processing.

(B) Local resolution estimation of the final cryo-EM density map showing better than 3 Å resolution for the majority of CNTFRα.

(C) FSC curve of the CNTFRα/REGN8938 Fab/H4H25322P2 Fab complex reconstruction showing a global resolution of 2.93 Å with the 0.143 gold standard threshold.

(D) Representative cryo-EM density of the complex. HC: heavy chain; LC: light chain.

(E) Colored density map and atomic model of the CNTFRα/REGN8938 Fab/H4H25322P2 Fab complex. HC: heavy chain; LC: light chain.

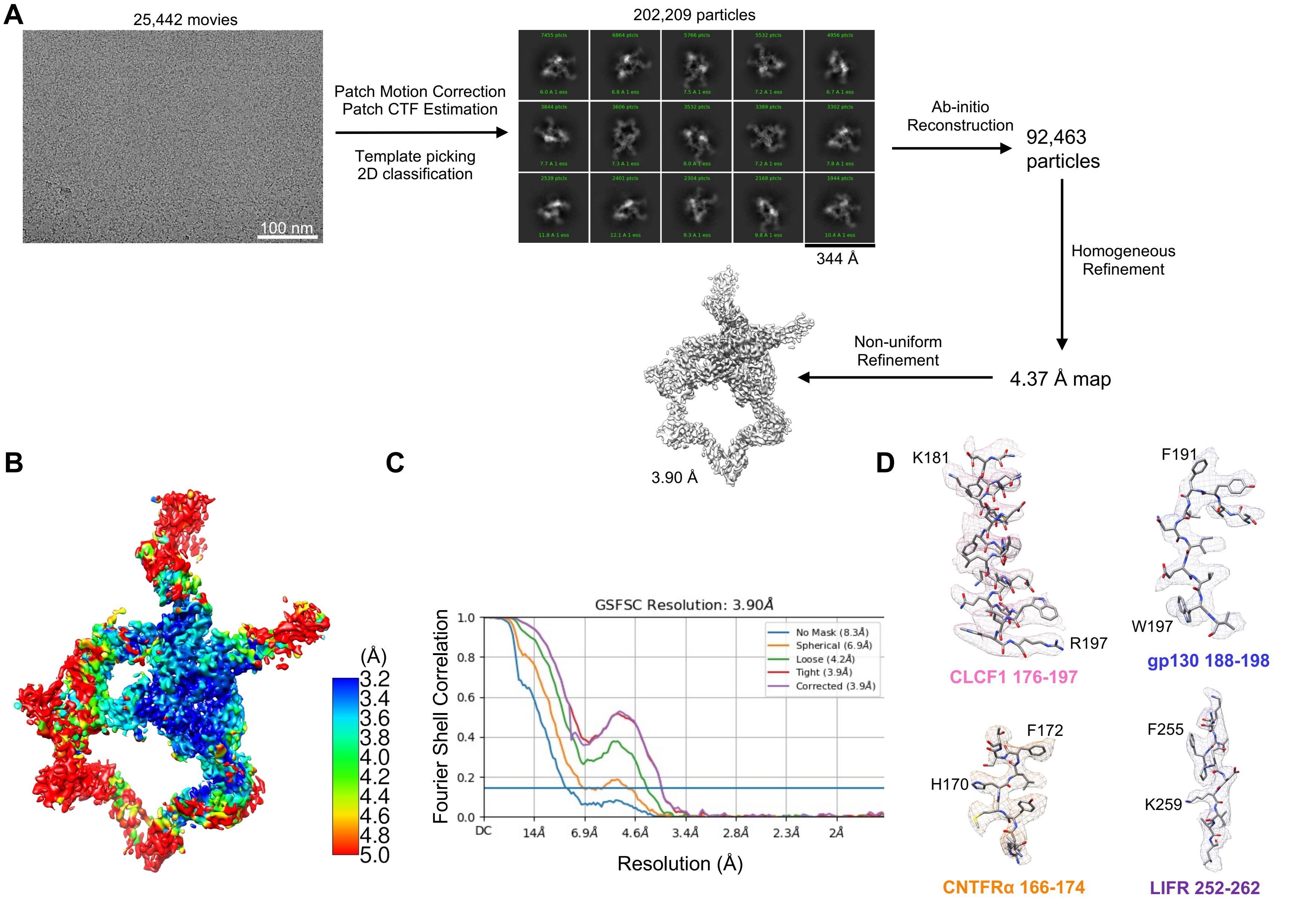

Fig. S4. Cryo-EM analysis of the CLCF1 signaling complex

(A) Workflow for cryo-EM data processing.

(B) Local resolution estimation of the final cryo-EM density map of the CLCF1 complex showing around 3.4 Å local resolution at the interaction core region, including CLCF1, CNTFRα D2D3, gp130 D2-D5, and LIFR D2-D5.

(C) FSC curve of the CLCF1 complex reconstruction showing a global resolution of 3.90 Å with the 0.143 gold standard threshold.

(D) Representative cryo-EM density of the 3D reconstruction in (B).

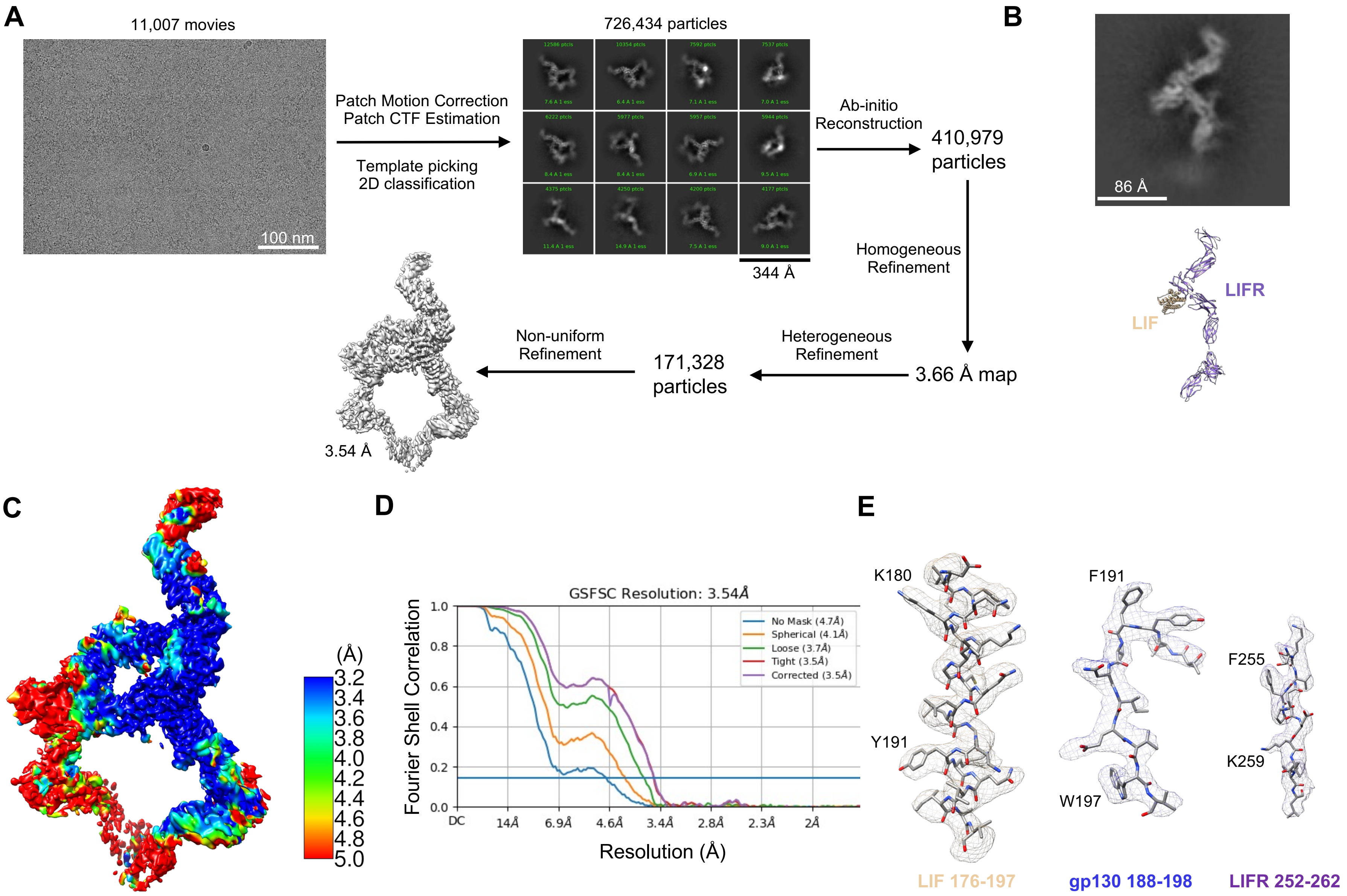

Fig. S5. Cryo-EM analysis of the LIF signaling complex

(A) Workflow for cryo-EM data processing.
(B) LIF complex intermediate (LIF/LIFR sub-complex) observed on the EM grid.
(C) Local resolution estimation of the final cryo-EM density map showing around 3.2 Å local resolution at the interaction core region, including LIF, gp130 D2-D5, and LIFR D2-D5.
(D) FSC curve of the LIF complex reconstruction showing a global resolution of 3.54 Å with the 0.143 gold standard threshold.
(E) Representative cryo-EM density of the 3D reconstruction in (C).

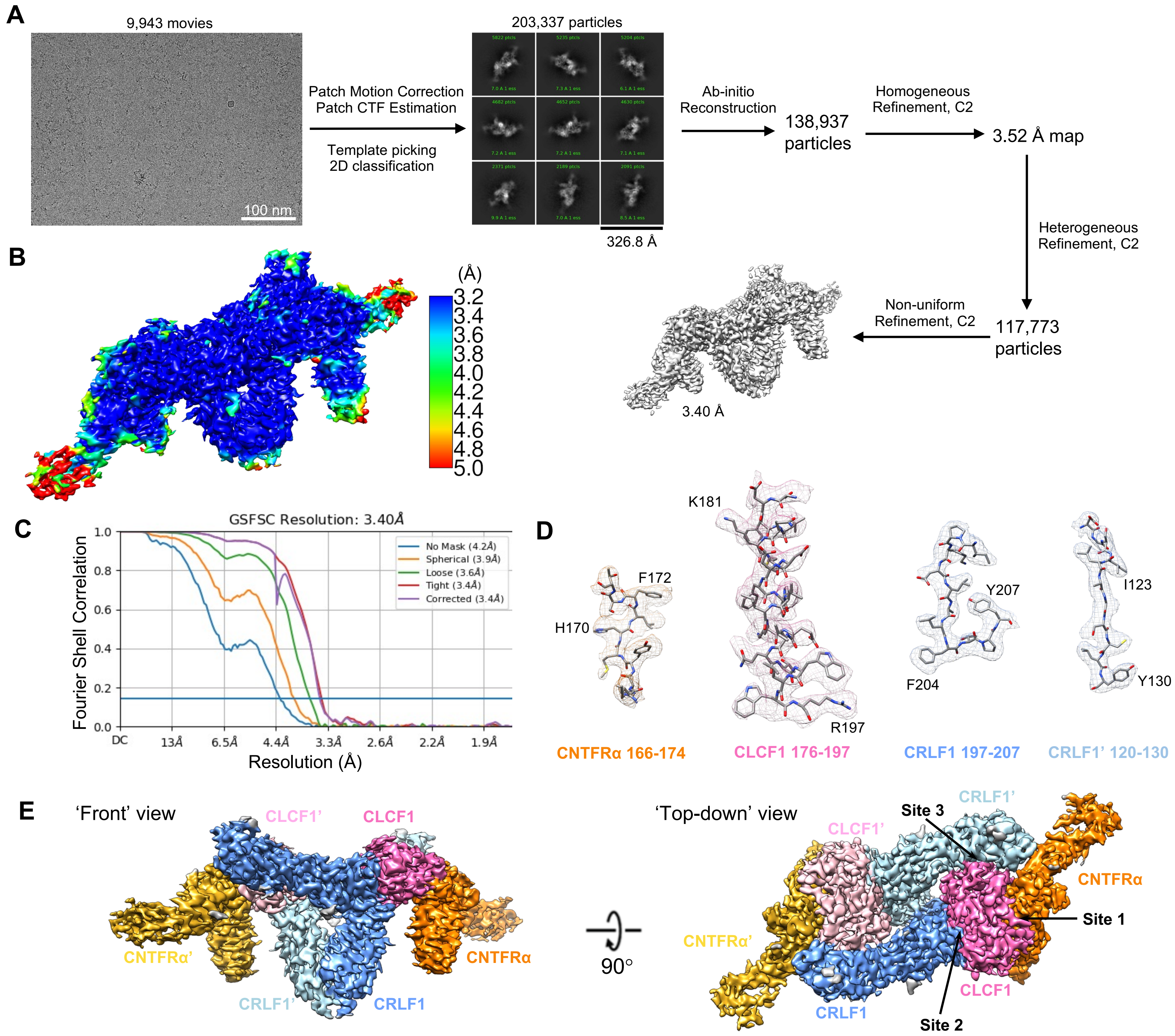

Fig. S6. Cryo-EM analysis of the CRLF1-CLCF1-CNTFRα complex

(A) Workflow for cryo-EM data processing.
(B) Local resolution estimation of the final cryo-EM density map of the CRLF1-CLCF1-CNTFRα complex.
(C) FSC curve of the CRLF1-CLCF1-CNTFRα complex reconstruction showing a global resolution of 3.40 Å with the 0.143 gold standard threshold.
(D) Representative cryo-EM density of the 3D reconstruction in (B).
(E) Colored density map of the CRLF1-CLCF1-CNTFRα complex in 'front' and 'top-town' views showing 2-fold symmetry of the complex. The two sets of molecules in the hexameric complex are annotated as CRLF1, CLCF1, CNTFRα, CRLF1', CLCF1', and CNTFRα'.

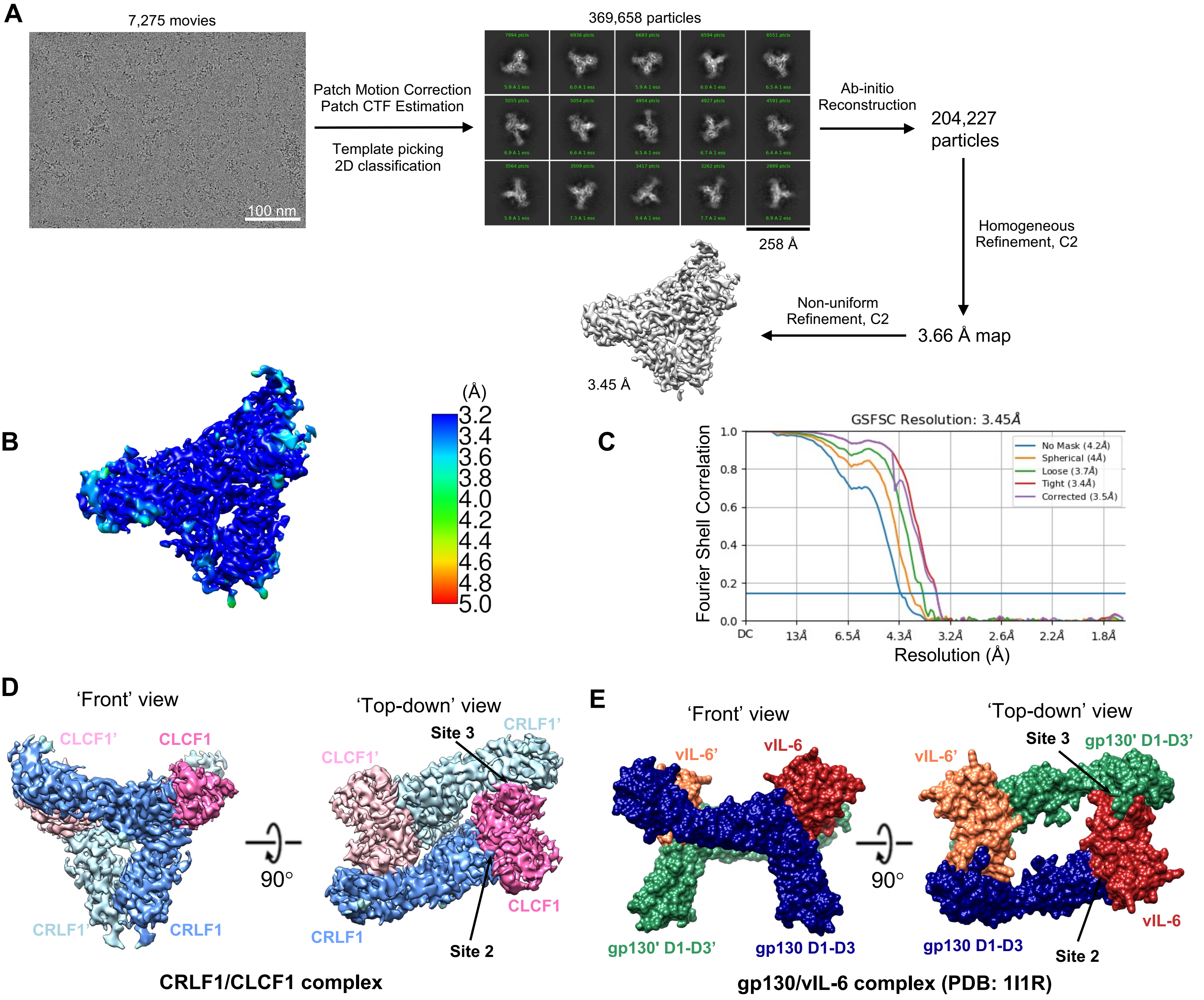

Fig. S7. Cryo-EM analysis of the CRLF1-CLCF1 complex

(A) Workflow for cryo-EM data processing.
(B) Local resolution estimation of the final cryo-EM density map of the CRLF1-CLCF1 complex .
(C) FSC curve of the CRLF1-CLCF1 complex reconstruction showing a global resolution of 3.45 Å with the 0.143 gold standard threshold.
(D) Colored density map of the CRLF1-CLCF1 complex in 'front' and 'top-town' views showing 2-fold symmetry of the complex. The two sets of molecules in the tetrameric complex are annotated as CRLF1, CLCF1, CRLF1', and CLCF1'.
(E) Surface representation of the gp130 D1-D3/vIL-6 complex crystal structure (PDB: 1I1R) in 'front' and 'top-town' views showing similar architectures of this complex and the CRLF1-CLCF1 complex.

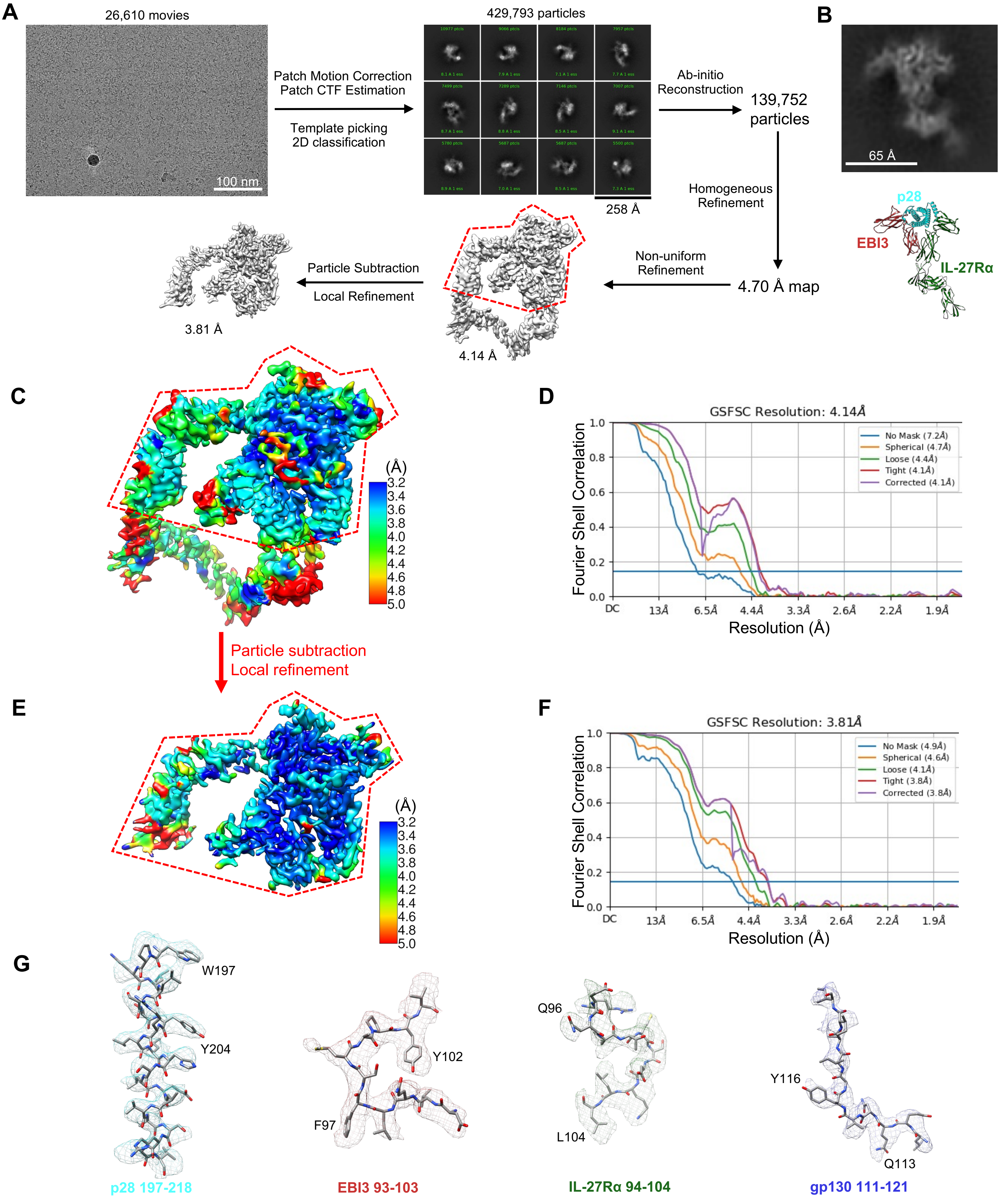

Fig. S8. Cryo-EM analysis of the IL-27 signaling complex

(A) Workflow for cryo-EM data processing.
(B) IL-27 complex intermediate (p28/EBI3/IL-27Rα sub-complex) observed on the EM grid.
(C) Local resolution estimation of the final cryo-EM density map showing around 3.6 Å local resolution at the interaction core region. This map was used to build a complete model of the IL-27 signaling complex. Models of gp130 D4-D6 derived from PDB:3L5H and IL-27Rα D3-D5 predicted by AlphaFold were rigid-body refined against the EM density. The map and model were used to show the overall architecture of the complex as shown in Figs. 4A-4C.
(D) FSC curve of the full IL-27 complex reconstruction showing a global resolution of 4.14 Å with the 0.143 gold standard threshold.
(E) Particles from (C) was subjected to particle subtraction and focused refinement around the interaction core region, including p28, EBI3, IL-27Rα D1D2, and gp130 D1-D3. Local resolution estimation of the focused refinement map shows around 3.4 Å local resolution at the binding interfaces. This map was used to build the atomic model for the interaction core region. The model was used to analyze the interaction details as shown in Figs. 5A-5D.
(F) FSC curve of the focused refinement map showing a resolution of 3.81 Å with the 0.143 gold standard threshold.
(G) Representative cryo-EM density of the 3D reconstruction in (E).

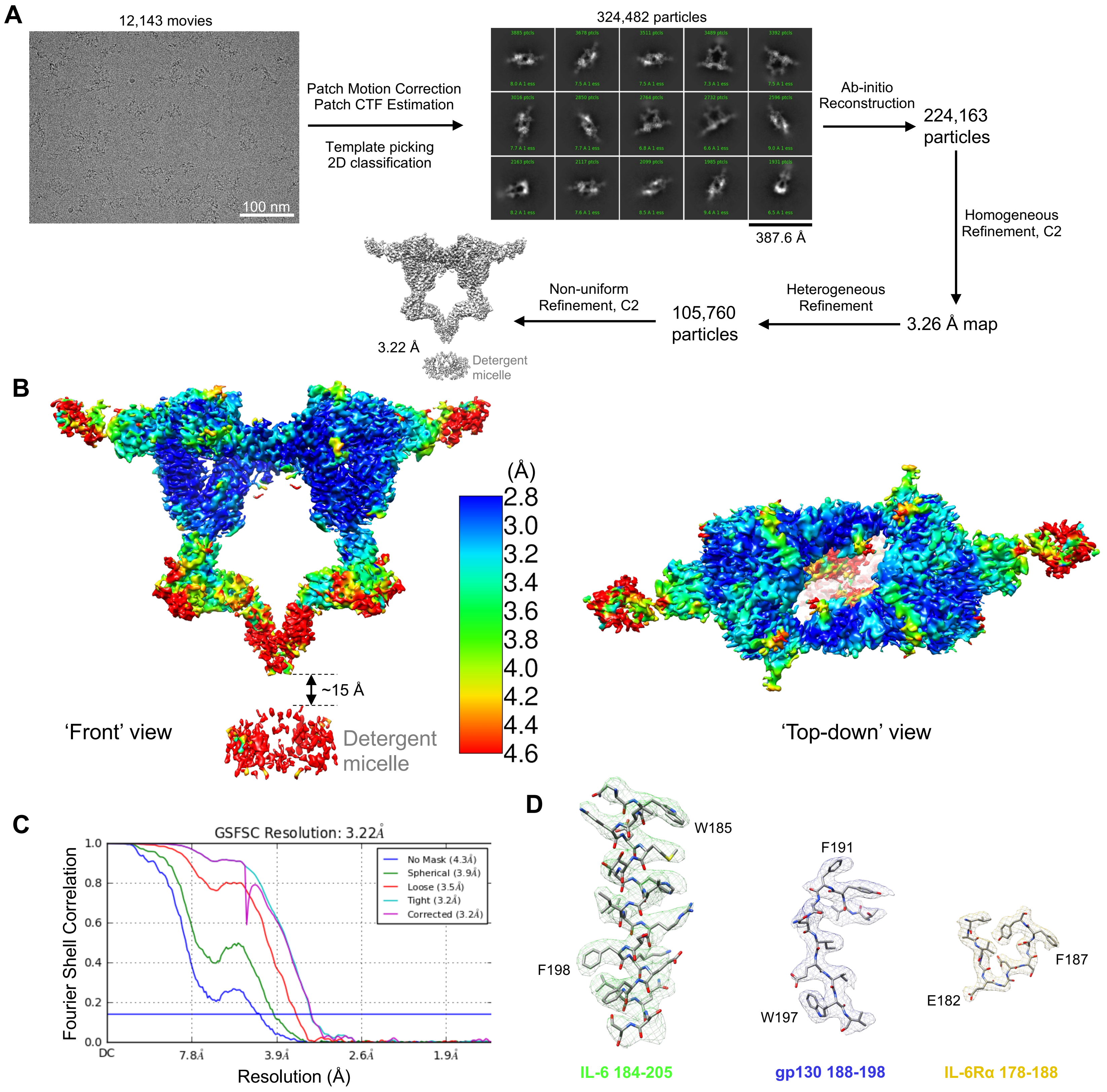

Fig. S9. Cryo-EM analysis of the IL-6 signaling complex in detergent

(A) Workflow for cryo-EM data processing.
(B) Local resolution estimation of the final cryo-EM density map in 'front' and 'top-town' views showing around 2.8 Å local resolution at the interaction core region and 2-fold symmetry of the complex. The TM domain of gp130 is not resolved in the detergent micelle due to flexibility. There is a ~15 Å gap between gp130 C-terminal density and the detergent micelle, which likely represents the flexible linker between gp130 D6 and TM region.
(C) FSC curve of the IL-6 complex reconstruction showing a global resolution of 3.22 Å with the 0.143 gold standard threshold.
(D) Representative cryo-EM density of the 3D reconstruction in (B).

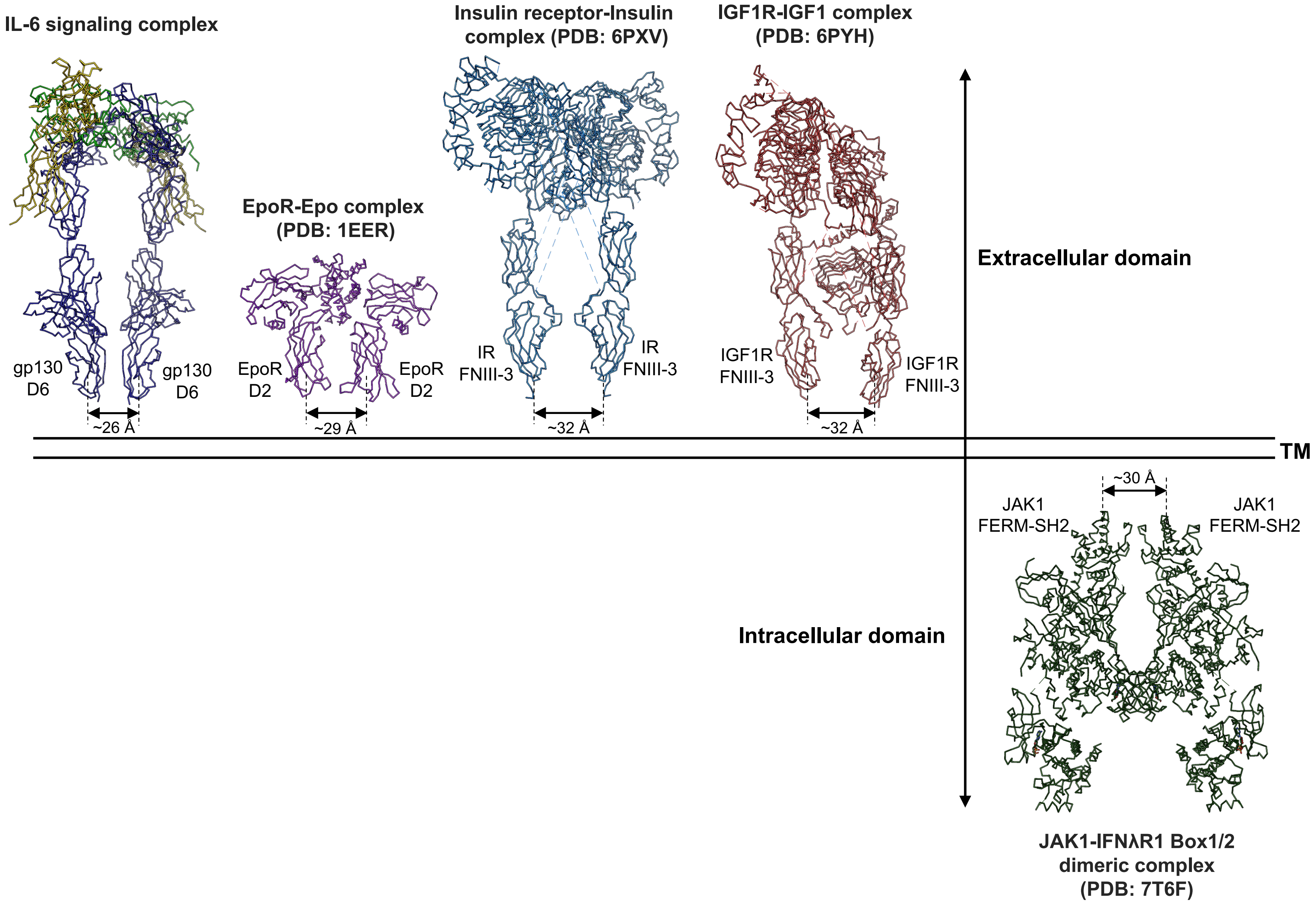

Fig. S10. Comparison of distances between membrane-proximal domains in various cytokine signaling complexes and the intracellular JAK1-IFNλR1 dimeric complex

The distances between the bottom centers of the membrane-proximal domains of the two signaling receptors in the IL-6 complex, EpoR-Epo complex (PDB: 1EER), Insulin receptor-insulin complex (PDB: 6PXV), IGF1R-IGF1 complex (PDB: 6PYH) are all around 30 Å. Moreover, on the intracellular side, the distance between the two membrane-proximal FERM-SH2 domains in the JAK1-IFNλR1 dimeric complex (PDB: 7T6F) is also about 30 Å.

Table S1. Sequence identity scores (%) among seven gp130 family cytokines calculated using Gonnet similarity matrix in MacVector software

	CNTF	CLCF1	LIF	IL-27 p28	IL-6	OSM	IL-11
**CNTF**	100.0	15.7	11.5	14.9	8.4	9.5	8.9
**CLCF1**	15.7	100.0	12.6	17.7	11.1	12.5	16.8
**LIF**	11.5	12.6	100.0	6.0	12.1	8.7	11.3
**IL-27 p28**	14.9	17.7	6.0	100.0	14.1	9.5	19.8
**IL-6**	8.4	11.1	12.1	14.1	100.0	10.0	14.7
**OSM**	9.5	12.5	8.7	9.5	10.0	100.0	11.0
**IL-11**	8.9	16.8	11.3	19.8	14.7	11.0	100.0

Table S2. Cryo-EM data collection, processing, and refinement statistics

	**CNTF signaling complex**		**CLCF1 signaling complex**	**LIF signaling complex**	**IL-27 signaling complex**		**IL-6 signaling complex**	**CRLF1-CLCF1-CNTFRα complex**	**CNTFRα-REGN8938 Fab-H4H25322P2 Fab complex**
**Data collection and processing**									
Magnification	105,000		105,000	105,000	105,000		105,000	105,000	105,000
Voltage (kV)	300		300	300	300		300	300	300
Electron exposure (e^-/Å^2)	40		40	40	40		40	40	40
Defocus range (μm)	-1.4 to -2.6		-1.4 to -2.6	-1.4 to -2.6	-1.4 to -2.6		-1.4 to -2.6	-1.4 to -2.6	-1.4 to -2.6
Pixel size (Å)	0.85		0.86	0.86	0.86		0.85	0.86	0.85
Number of movies	6,511		25,442	11,007	26,610		12,143	9,943	7,859
Initial number of particles	3,197,974		13,119,612	5,545,456	19,480,836		3,881,621	1,258,062	4,515,671
Final selected particles	250,735	100,013	92,463	171,328	139,752		105,760	117,773	568,328
Symmetry imposed	C1		C1	C1	C1		C2	C2	C1
Map resolution (Å)	3.03	3.37	3.90	3.54	3.81	4.14	3.22	3.40	2.93
FSC threshold	0.143		0.143	0.143	0.143		0.143	0.143	0.143
**Refinement**									
Map sharpening *B* factor (Å^2)	-70	-49.6	-90	-51.4	-120	-90	-78	-94.5	-99.6
Model composition									
Non-hydrogen atoms	9,640	-	9,628	7,912	7,101	-	17,366	12,542	8,960
Protein residues	1,163	-	1,167	963	865	-	2,108	1,526	1,143
Ligands	18	-	16	15	11	-	38	32	8
R.m.s. deviations									
Bond lengths (Å)	0.002	-	0.002	0.002	0.002	-	0.002	0.002	0.002
Bond angles (°)	0.448	-	0.486	0.507	0.471	-	0.454	0.449	0.447
Model vs. map									
CC (volume)	0.76	-	0.54	0.71	0.63	-	0.75	0.73	0.75
CC (mask)	0.79	-	0.56	0.73	0.65	-	0.76	0.76	0.78
Mean Protein *B* factor (Å^2)	81.75	-	55.58	87.06	60.19	-	85.93	85.34	28.43
Validation									
MolProbity score	1.66	-	1.79	1.85	1.55	-	1.43	1.34	1.53
Rotameric outliers (%)	0.85	-	0.00	0.00	0.00	-	0.95	1.06	1.30
Clash score	5.34	-	5.56	6.62	4.91	-	3.18	2.71	5.45
Ramachandran plot									
Favored (%)	94.54	-	91.88	91.95	95.79	-	95.37	96.10	97.17
Allowed (%)	5.46	-	8.12	8.05	4.09	-	4.63	3.90	2.83
Disallowed (%)	0.00	-	0.00	0.00	0.12	-	0.00	0.00	0.00
**Deposition ID**									
PDB	8D74	-	8D7R	8D6A	8D85	-	8D82	8D7H	8D7E
EMDB	27227[a]	27229[b]	27231	27221	27247[c]	27246[d]	27244	27230	27228

[a] Cryo-EM map of the CNTF signaling complex: refined from the selected full particle dataset, with better resolution in the interaction core region

[b] Cryo-EM map of the CNTF signaling complex: refined from a particle subset, with lower global resolution but improved LIFR D6-D8 density

[c] Cryo-EM map of the IL-27 signaling complex: focused refinement on the interaction core region

[d] Cryo-EM map of the IL-27 signaling complex with full ECDs
